# Comment on “Evidence for Negative Effects of Elevated Intra-Abdominal Pressure on Pulmonary Mechanics and Oxidative Stress”

**DOI:** 10.1155/2015/746937

**Published:** 2015-06-11

**Authors:** Almas K. Ormantayev, Anar D. Sepbayeva, Ioannis P. Kosmas, Amirkhan K. Baimaganbetov, Viktor Y. Issakov, Ospan A. Mynbaev

**Affiliations:** ^1^Department of Pediatric Surgery, Kazakh National Medical University, Tolebi Street 94, Almaty 050000, Kazakhstan; ^2^Moscow Institute of Physics and Technology (State University), Institutskii Lane 9, Dolgoprudny, Moscow 141700, Russia; ^3^Department of Obstetrics and Gynecology, Ioannina State General Hospital “G. Chatzikosta”, 45001 Ioannina, Greece; ^4^Department of Surgical Disciplines and Cardiovascular Surgery, Faculty of Medicine, Ahmet Yesevi International Kazakh-Turkish University, Zhandosova Street 92, Shymkent 486035, Kazakhstan; ^5^Department of Obstetrics, Gynecology and Reproductive Medicine, Peoples' Friendship University of Russia, Miklukho-Maklay Street 21/3, Moscow 117198, Russia; ^6^The New European Surgical Academy, Unter den Linden 21, 10117 Berlin, Germany

We read with great interest the recently published article by Davarcı et al. [1] in your journal aimed at studying the effects of CO_2_-pneumoperitoneum at 12 mm Hg intraperitoneal pressure on end-tidal CO_2_ (P_ET_CO_2_) concentration, arterial blood gas values and oxidative stress markers in blood, and bronchial lavage during laparoscopic cholecystectomy using a long protective strategy since our clinical [2] and experimental [3] results were in line with findings of this study [1]. The authors clearly demonstrated significant changes of the peak in respiratory pressure, dynamic lung compliance, P_ET_CO_2_, arterial pO_2_, pCO_2_, and pH values at the 30th min of CO_2_-pneumoperitoneum in comparison with parameters of both at the baseline and at the end of surgery. These changes we considered as consequences of a causative force of CO_2_-insufflation with increased content of CO_2_ in the body (rise of P_ET_CO_2_ and arterial pCO_2_), with subsequent mild respiratory or severe acidosis (reduced pH) depending on intraperitoneal pressure rate and CO_2_-pneumoperitoneum duration [4–6]. Subsequently, the dynamic lung compliance was reduced with increased peak of respiratory pressure in adult patients with ASA I/II [1].

We have monitored respiratory and cardiovascular parameters (systolic/diastolic arterial pressure, heart rate, cardiac output, ventilation rate and pressure, tidal volume, and P_ET_CO_2_), the dynamic lung compliance, the peak in respiratory pressure, skin temperature, and urine output with catheter in 12 newborns suffering laparoscopic surgical procedures due to ovarian tumors [2]. All samples were collected at the time of induction, at the time of incision, and every 10 minutes during surgery and after surgery during one and a half hours, subsequently at the eleven time points (0–10). All babies were born at the full term pregnancies with body weight above 3000 g. Anesthesia was induced by Relanium or Midazolam (0,63 ± 0,27 mg/kg/h) and Fentanyl (11,9 ± 5,8 *μ*g/kg/h); pressure controlled mechanical ventilation was done by means of anesthesia-respiratory ventilator (Drager) supplemented with myorelaxants (cisatracurium besilate 0,14 ± 0,05 mg/kg/h or Atracurium 0,54 ± 0,19 mg/kg/h).

In newborns during laparoscopic surgery, P_ET_CO_2_ value was significantly increased (Figure 1(a)) during the first 20 minutes of CO_2_-pneumoperitoneum at the 7–9 mm Hg of intraperitoneal pressure, which was corrected by mild hyperventilation with increased ventilation rate (VR). These changes were accompanied with increased systolic and diastolic arterial blood pressure and decreased cardiac output [2]. Moreover, such parameters as respiratory volume, minute ventilation rate, and dynamic lung compliance were reduced with increased peak of respiratory pressure, whereas heart rate, urine output, and skin temperature were remaining stable [2].

In our experimental studies, all parameters of blood gases, acid base homeostasis, blood oximetry, and oxygen status were monitored in anesthetized and ventilated rabbits as control group, and spontaneously breathing (series I) and superficially (series II) either optimally (series IIIA) ventilated animals with intraperitoneal CO_2_-insuflation at 10 mmHg including an additional subseries with 6 mmHg in optimally ventilated animals (series IIIB), as experimental groups [3]. Changes in blood gases, acid base parameters were clearly shown (Figure 1(b)) during CO_2_-pneumoperitoneum at two levels of intraperitoneal pressure (6 and 10 mm Hg) in different ventilation modes in rabbits, which is an appropriate model for newborns.

It is well known that CO_2_-pneumoperitoneum is associated with carboxemia, acidemia, acidosis, and base deficiency with changes in oxygen metabolism, which was suggested as metabolic hypoxemia [3].

Results of these studies [1–3] clearly demonstrated negative effects of elevated intraperitoneal pressure on parameters of blood gases, acid base, and oxygen homeostasis as well as respiratory and cardiovascular systems during laparoscopic procedures. Obviously, these changes were pronounced in newborns in relatively lower intraperitoneal pressure (7–9 mm Hg). Analogously in 40 adult patients who experienced laparoscopic transabdominal preperitoneal and extraperitoneal inguinal hernia repair, Zhu et al. [7] observed CO_2_ accumulation, acidosis, increased blood pressure, and decreased heart rate, which were controlled by appropriate treatments during the operation, whereas Hypolito et al. [8] demonstrated higher disturbances in mean arterial pressure, pCO_2_, pH, HCO_3_, and base excess in 37 patients during CO_2_-pneumoperitoneum at 20 mm Hg in comparison with mild transient changes without their clinical manifestations in 30 patients during CO_2_-pneumoperitoneum at 12 mm Hg.

Findings of these studies supporting correlations of changes in arterial blood gases with the end-tidal CO_2_ concentration, as well as ventilation parameters and dynamic lung compliance [1–3], were in accordance with results by Strang et al. [9] concerning arterial pCO_2_ to end-tidal CO_2_ gradient, which is strongly correlated with the amount of atelectasis estimated by an end-expiratory transversal spiral computed tomography with subsequent calculation of the total lung volume with further analysis of the lung tissue density as normally, poorly, over-, and nonaerated (atelectasis) regions.

Recently, in two similar prospective studies, Oksar et al. [10, 11] monitored blood gas and end-tidal CO_2_ values and hemodynamic parameters (heart rate, mean arterial, and central venous pressures) affected by CO_2_-pneumoperitoneum alone and in combination with Trendelenburg position and concluded that the main challenges associated with these conditions were the respiratory acidosis and “upper airway obstruction-like” clinical symptoms.

In order to widely apply laparoscopic procedures in pediatric surgery, we should take into account an increased intracranial pressure during CO_2_-pneumoperitoneum with steep Trendelenburg positioning (30°) proven by ultrasonographic measurement of optic nerve sheath diameter observed in 20 patients who underwent elective robot-assisted laparoscopic radical prostatectomy with an intra-abdominal pressure of 15 mm Hg [12]. Moreover, these findings were proved in experimental study by a strong correlation of increased intracranial pressure with corresponding intraperitoneal (intravesical) pressure in six female pigs [13]. Subsequently, surgeons should be aware about these side effects of CO_2_-pneumoperitoneum which can be pronounced especially in pediatric patients.

In conclusion, it should be a concern in an upcoming era of worldwide increased application of robotic tools and laparoscopic surgical procedures in all categories of patients including children taking into account possible side effects of CO_2_-pneumoperitoneum.

## Figures and Tables

**Figure 1:**
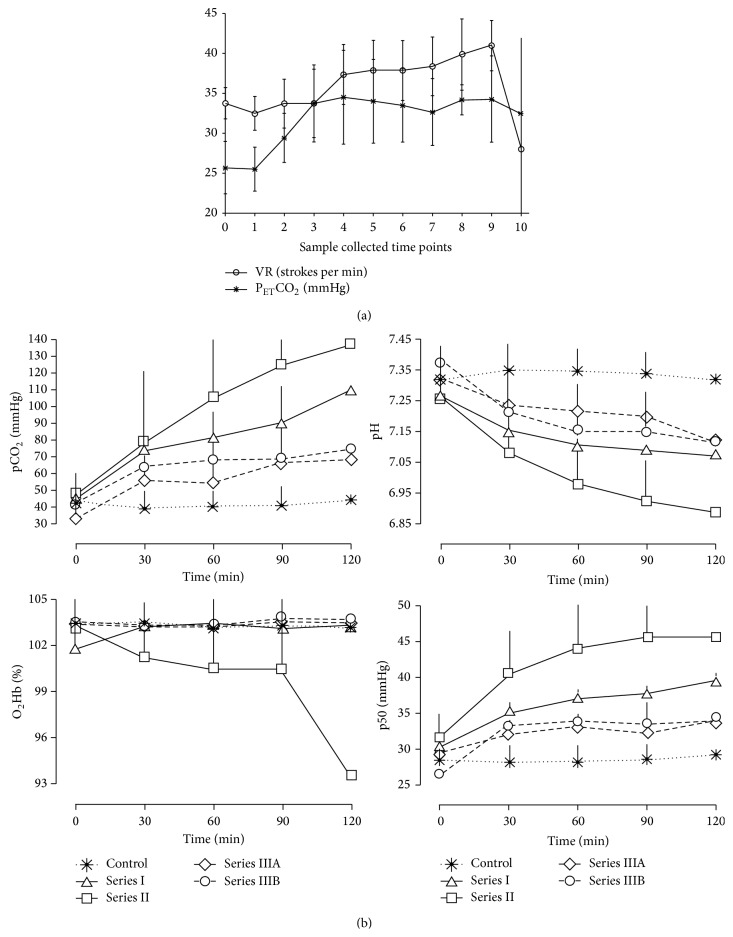
An impact of CO_2_-pneumoperitoneum on respiratory, blood gases, and oxygen status parameters: (a) the end-tidal CO_2_ concentration (P_ET_CO_2_) and ventilation rate (VR) parameters in 12 newborns, suffering laparoscopic surgical procedures due to ovarian tumors, at the time of induction (0), at the time of incision (1), and every 10 minutes during (2–9) and after (10) laparoscopic surgery with CO_2_-pneumoperitoneum at 7–9 mmHg (eleven sampling points) from [2], unpublished data); (b) an arterial blood carbon dioxide partial pressure (pCO_2_), pH, oxyhemoglobin (O_2_Hb), and oxygen tension at half saturation assessing the hemoglobin oxygen affinity (p50): in rabbits without pneumoperitoneum (control), spontaneously breathing animals (series I), superficially ventilated animals (series II), and optimally ventilated animals with insufflation pressures of 10 mmHg (series IIIA) or 6 mmHg (series IIIB). Values are means ± SD (modified from [3]).
